# Single Nucleotide Polymorphisms in RUNX2 and BMP2 contributes to different vertical facial profile

**DOI:** 10.1371/journal.pone.0303551

**Published:** 2024-05-21

**Authors:** Caio Luiz Bitencourt Reis, Mirian Aiko Nakane Matsumoto, Maria Bernadete Sasso Stuani, Fábio Lourenço Romano, Rafaela Scariot, Angela Graciela Deliga Schroder, Paulo Nelson-Filho, Christian Kirschneck, Svenja Beisel-Memmert, Erika Calvano Küchler

**Affiliations:** 1 Department of Pediatric Dentistry, School of Dentistry of Ribeirão Preto, University of São Paulo, São Paulo, Brazil; 2 Department of Stomatology, Federal University of Paraná, Curitiba, Brazil; 3 School of Dentistry, Tuiuti University of Paraná, Curitiba, PR, Brazil; 4 Department of Orthodontics, University Hospital Bonn, Medical Faculty, Bonn, Germany; University of Catania: Universita degli Studi di Catania, ITALY

## Abstract

The vertical facial profile is a crucial factor for facial harmony with significant implications for both aesthetic satisfaction and orthodontic treatment planning. However, the role of single nucleotide polymorphisms (SNPs) in the development of vertical facial proportions is still poorly understood. This study aimed to investigate the potential impact of some SNPs in genes associated with craniofacial bone development on the establishment of different vertical facial profiles. Vertical facial profiles were assessed by two senior orthodontists through pre-treatment digital lateral cephalograms. The vertical facial profile type was determined by recommended measurement according to the American Board of Orthodontics. Healthy orthodontic patients were divided into the following groups: “Normodivergent” (control group), “Hyperdivergent” and “Hypodivergent”. Patients with a history of orthodontic or facial surgical intervention were excluded. Genomic DNA extracted from saliva samples was used for the genotyping of 7 SNPs in *RUNX2*, *BMP2*, *BMP4* and *SMAD6* genes using real-time polymerase chain reactions (PCR). The genotype distribution between groups was evaluated by uni- and multivariate analysis adjusted by age (alpha = 5%). A total of 272 patients were included, 158 (58.1%) were “Normodivergent”, 68 (25.0%) were “Hyperdivergent”, and 46 (16.9%) were “Hypodivergent”. The SNPs rs1200425 (*RUNX2*) and rs1005464 (*BMP2*) were associated with a hyperdivergent vertical profile in uni- and multivariate analysis (p-value < 0.05). Synergistic effect was observed when evaluating both SNPs rs1200425- rs1005464 simultaneously (Prevalence Ratio = 4.0; 95% Confidence Interval = 1.2–13.4; p-value = 0.022). In conclusion, this study supports a link between genetic factors and the establishment of vertical facial profiles. SNPs in *RUNX2* and *BMP2* genes were identified as potential contributors to hyperdivergent facial profiles.

## Introduction

The vertical facial profile is strongly associated with the patient’s aesthetic satisfaction and self-esteem, which are powerful motivational factors for seeking orthodontic treatment [1, 2]. Patients with hyperdivergent facial profile exhibit an increased lower anterior face height compared to upper anterior face height, while patients with hypodivergent facial profile show the opposite pattern. Normodivergent patients have harmonious expression of the vertical proportions.

The development of the individual’s vertical facial pattern occurs during craniofacial growth [3, 4]. Craniofacial growth is a complex process which involves interactions between cells, proteins, and several genes. Various dysfunctions may affect craniofacial growth and disrupt healthy and coordinated growth in terms of timing, magnitude, and direction [5–8]. Several studies suggested that Single Nucleotide Polymorphisms (SNPs) in key genes may be involved in the development of different facial growth patterns [8–10]. SNPs are a type of genetic variation responsible for diversity among individuals. SNPs are characterized by the wild allele substitution, which may change the amino acid sequence of a protein or directly affect the quantity, quality, and stability of gene expression [11]. Although twin studies clearly indicate that the vertical growth pattern is strongly influenced by genetic factors [12], studies investigating the aetiology of skeletal facial growth patterns have been mainly focused on variations in anteroposterior dimensions of the face [8, 9]. The role of SNPs, however, in the development of vertical facial proportions is still poorly understood [7, 8].

Recent studies exploring SNPs in genes associated with bone physiology have gained attention due to the possible impact on craniofacial growth [8, 10, 13, 14]. *RUNX2* (Runt-related transcription factor 2) gene encodes the master bone transcription factor, crucial for osteoblast differentiation, driving skeletal development and important for craniofacial and dental morphogenesis in vertebrates [15]. RUNX2 is a downstream target of bone morphogenetic proteins (BMP) family members [16]. The BMP family also plays a key role in craniofacial development, particularly BMP2 and BMP4, which are important for osteoblast differentiation and bone mineralization [17]. This BMP-RUNX2 axis is negatively regulated by SMAD6, a member of the Smad (Suppressor of mother against decapentaplegic) protein family. This feedback mechanism regulates signaling pathways during bone development and maintenance [18]. SNPs in these genes have already been associated with anteroposterior skeletal malocclusions [8]. Therefore, we hypothesized that these SNPs could also be involved in the vertical growth of the face. In the present study, we investigated the association between different vertical facial profiles and SNPs in *RUNX2*, *BMP2*, *BMP4*, and *SMAD6*.

## Methods

The Research Ethics Committees at the School of Dentistry ************* (Protocol No. 50765715.3.0000.5419) and **************** (Protocol No. 80846317.8.0000.0093) granted approval for this study. This study was conducted in accordance with the principles outlined in the Declaration of Helsinki and its subsequent revisions. The researchers invited the patients and legal guardians of minor patients and provided a document containing the details of the study. Patients who agreed to participate in the study indicated their consent with a handwritten signature. Minor patients received an age-appropriate document and also indicated their consent with a handwritten signature, along with their legal guardians. All patients were capable of reading and writing.

### Study design

This retrospective cross-sectional study was designed throughout March 2023 and reported here through a specific genetic association studies guideline (STrengtheningthe REporting of Genetic Association Studies - STREGA): An Extension of the STROBE Statement [19].

### Study size

Sample size calculation was performed through G*Power software (Version 3.1.9.7, University of Kiel, Germany). The test “Contingency table” was applied with alpha set at 5%, power at 80%, and Degrees of Freedom at 1. The expected effect size, Cohen’s w (0.23), was obtained from the study by Cunha et al. [7] indicating a total requirement of 140 patients for this study.

### Setting and participants

Patients of both sexes, aged 8 years and older from two Brazilian universities’ orthodontic clinics in ********** (***** state) and ******** (******** state) were recruited between March 1, 2018 and October 31, 2021. Patients with uncontrolled systemic medical conditions, cleft lip and palate, a significant number of missing posterior or anterior teeth, or a history of facial trauma or previous orthodontic/orthognathic treatment were excluded.

### Variables and data sources measurement

**Fig 1:**
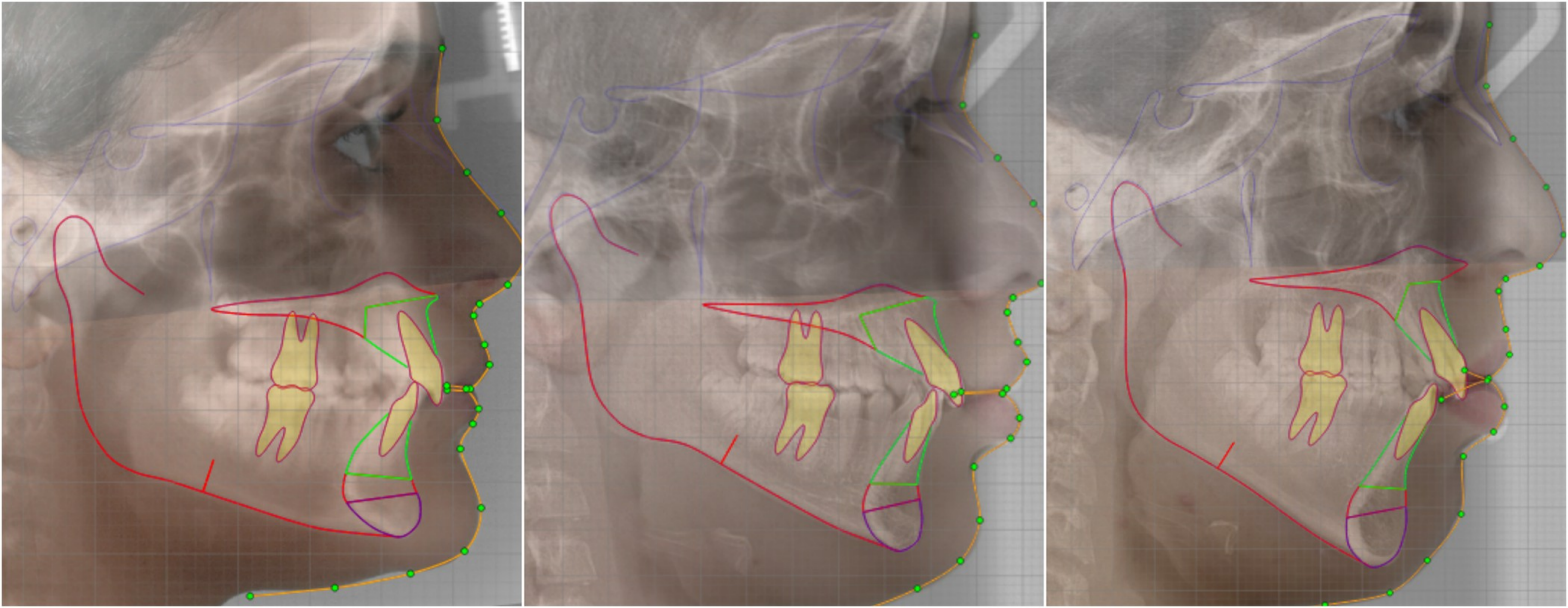
Representative images of three patients with different vertical facial profiles. The digital lateral cephalograms and profile pictures of each patient were merged. The yellow lines indicate the SN and MP lines. A: Hypodivergent profile. B: Normodivergent profile. C: Hyperdivergent profile.

The vertical facial profile was assessed retrospectively between April 1, 2023 and July 25, 2023 trough pre-orthodontic digital lateral cephalograms. The patients were diagnosed according to the criteria outlined by the American Board of Orthodontics (Discrepancy index, 2016). The angle formed between the Sella-Nasio (SN) line and the Mandibular Plane (MP) was obtained by two experienced orthodontists (more than 10 years of experience). Cephalometric tracings were conducted using Dolphin software (Chatsworth, CA, U.S.A). Patients with SN-MP values ranging from 27° to 37° were classified as having a normodivergent facial profile. Patients with SN-MP values below 27° were categorized as hypodivergent profile patients, while patients with SN-MP values exceeding 37° were classified as hyperdivergent profile patients (Fig 1).

Genomic DNA extraction from buccal epithelial cells in saliva samples initiated in March 10, 2019, and ended on January 15, 2022, as previously detailed [20]. The concentration and purity of the DNA were assessed using a spectrophotometer (Nanodrop 1000; Thermo Scientific, Wilmington, DE, USA). The SNPs were genotyped between March 20, 2019 and April 18, 2022 using real-time polymerase chain reactions (PCR) (StepOnePlus™ Real-time PCR System, Applied Biosystems, Foster City, CA, USA). Real-time PCR reactions were conducted in a total volume of 3 μl (4 ng DNA per reaction, 1.5 μl Taqman PCR master mix, 0.075 SNP assay; Applied Biosystems, Foster City, CA, USA). The thermal cycling began with a hold cycle at 95°C for 10 min, followed by 40 amplification cycles of 92°C for 15 s and 60°C for 1 min.

Seven SNPs in the candidate genes related to craniofacial bone development (*RUNX2*, *BMP2*, *BMP4* and *SMAD6*) and previously associated with skeletal malocclusions [8] were selected for analysis. The details of the SNPs are shown in Table 1.

**Table 1:** Detailed information about the studied SNPs.

Gene	SNP	Chromosome	Wild Allele	Minor Allele	Minor allele frequency (%)*	Most severe consequence**	Genotyping success rate (%)	Hardy Weinberg χ ***
RUNX2	rs1200425	6	G	A	41.9	Intron Variant	91.2	1.30
	rs59983488	6	G	T	16.2	Intron Variant	89.3	0.44
BMP2	rs1005464	20	G	A	20.5	Intron Variant	92.3	0.88
	rs235768	20	T	A	30.8	Missense Variant	91.9	1.21
BMP4	rs17563	14	A	G	40.4	Missense Variant	91.9	0.00
SMAD6	rs3934908	4	C	T	48.6	Intron Variant	92.3	0.18
	rs2119261	15	C	T	39.4	Intron Variant	91.9	3.25

Notes

* In this study.

** According to Ensembl Project (ensembl.org).

*** Test eaxecuted by wpcalc.com.

### Bias

The inter-rater reliability for cephalometric analysis was assessed by Intraclass Correlation Coefficient test (0.80) with 20 patients not included in this study. The information of age and sex in digital lateral cephalograms were kept hidden from the orthodontists. Genotyping and statistical analysis were also blindly conducted to mitigate potential bias. We also adjusted the genotype distribution results for age.

### Statistical methods

Chi-square test was applied to evaluate SNPs Hardy-Weinberg equilibrium (Table 1).

The allele (Major vs. Minor) and genotype distribution of the SNPs were compared between “Normodivergent” vs “Hypodivergent” or “Normodivergent” vs “Hyperdivergent” groups. The genotype comparison was performed per models: Dominant (Homozygous common vs. Heterozygous + Homozygous uncommon) and Recessive (Homozygous common + Heterozygous vs. Homozygous uncommon).

Pearson’s chi-square without correction or Fisher exact tests were used in univariate models. Poisson regression was applied to multivariate models adjusted by age. Prevalence ratio (PR) with 95% Confidence Interval (CI) were calculated for multivariate models. SNP-SNP interaction was assessed in a multivariate model according to Küchler et al. [21].

The tests were carried out using IBM SPSS Statistics for Windows (Version 25.0. Armonk, NY: IBM Corp.), with alpha set at 5%.

## Results

A total of 272 patients were included in this study, 158 (58.1%) classified as “Normodivergent”, 68 (25.0%) classified as “Hyperdivergent”, and 46 (16.9%) classified as “Hypodivergent”. The age ranged from 8 to 58 years. A total of 112 individuals were male, 160 were female. More information about the sample is detailed in Table 2. Gender and age were not statistically different among groups (p > 0.05).

**Table 2:** Characteristics of sample per vertical facial profile.

Variables		Total		Normodivergent		Hyperdivergent		Hypodivergent	
		n	%	n	%	n	%	n	%
Total		272	100	158	58.09	68	25.00	46	16.91
Sex	Male	112	41.18	61	38.61	26	38.24	25	54.35
	Female	160	58.82	97	61.39	42	61.76	21	45.65
Age	Mean (SD)	22.14 (11.34)		21.51 (11.25)		22.57 (10.91)		23.65 (12.30)	

Note: SD means Standard deviation

The genotyping success rate for each studied SNP is described in Table 1. All SNPs were in Hardy-Weinberg equilibrium.

The allele and genotype distribution between groups in univariate models are shown in Table 3. The rs1200425 in *RUNX2* was associated with hyperdivergent facial profile in allelic (p = 0.035) and recessive models (p = 0.002). The rs1005464 in *BMP2* was associated with a hyperdivergent facial profile in a recessive model (p = 0.036).

**Table 3:** Frequency distribution table per genotypes and vertical facial profiles with univariate statistics.

Gene	SNP	Genotype	Normodivergent		Hyperdivergent		p-values per model			Hypodivergent		p-values per model		
			n	%	n	%	Allelic	Dominant	Recessive	n	%	Allelic	Dominant	Recessive
RUNX2	rs1200425	GG	52	36.1	20	31.7	0.035*	0.544	0.002*	16	39.0	0.824	0.732	0.375
		AG	72	50.0	23	36.5				17	41.5			
		AA	20	13.9	20	31.7				8	19.5			
	rs59983488	GG	100	71.4	37	60.7	0.145	0.131f	0.634f	32	76.2	0.431	0.980f	-
		GT	37	26.4	22	36.1				10	23.8			
		TT	3	2.1	2	3.3				0	0.0			
BMP2	rs1005464	GG	91	62.8	46	73.0	0.679	0.151	0.036*	24	55.8	0.266	0.411	0.199
		AG	50	34.5	11	17.5				16	37.2			
		AA	4	2.8	6	9.5				3	7.0			
	rs235768	TT	62	42.8	32	51.6	0.241	0.241f	0.470f	22	51.2	0.859	0.330	0.201
		AT	72	49.7	27	43.5				15	34.9			
		AA	11	7.6	3	4.8				6	14.0			
BMP4	rs17563	AA	50	34.2	21	33.9	0.995	0.958	0.956	18	42.9	0.489	0.306	0.972
		AG	72	49.3	31	50.0				17	40.5			
		GG	24	16.4	10	16.1				7	16.7			
SMAD6	rs3934908	CC	39	27.1	17	27.0	0.745	0.988	0.604	12	27.3	0.929	0.980	0.903
		CT	71	49.3	29	46.0				22	50.0			
		TT	34	23.6	17	27.0				10	22.7			
	rs2119261	CC	46	31.9	23	36.5	0.962	0.512	0.424	16	37.2	0.650	0.519	0.974
		CT	81	56.3	30	47.6				22	51.2			
		TT	17	11.8	10	15.9				5	11.6			

Note: Pearson’s chi-square test without correction was used, except for p-values with f, which was obtained by Fisher exact test. Fisher test was used when one of the expected cell count is less than 5. When an observed cell count is equal to 0, none test was performed.

* indicate statistical significance (p<0.05)

In the multivariate analysis adjusted by age (Table 4), the recessive genotype AA of the SNP rs1200425 in *RUNX2* increased the risk of hyperdivergent profile development (PR = 1.95; 95% CI 1.25–3.05; p = 0.003). The recessive genotype AA of the rs1005464 in *BMP2* also increased the risk of hyperdivergent profile development (PR = 2.07; 95% CI = 1.02–4.21). When the recessives genotypes of rs1200425 SNP in *RUNX2* (AA) and rs1005464 SNP in *BMP2* (AA) were evaluated together (SNP-SNP interaction), a synergism was observed (PR = 4.06; 95% CI = 1.22–13.47; p = 0.022).

**Table 4:** Summary of multivariate statistics results to hyperdivergent facial profile.

SNPs	Model	p-value	PR	95% CI	
				Lower	Upper
rs1200425 (*RUNX2)*	Allelic (G vs. A)	0.052	1.36	0.99	1.86
	Dominant (GG vs. AG + AA)	0.571	1.13	0.72	1.77
	Recessive (GG + AG vs. AA)	0.003*	1.95	1.25	3.05
rs1005464 (*BMP2*)	Allelic (G vs. A)	0.721	0.92	0.61	1.40
	Dominant (GG vs. AG + AA)	0.179	0.72	0.45	1.15
	Recessive (GG + AG vs. AA)	0.043*	2.07	1.02	4.21
Interaction between rs1200425 (*RUNX2) +* rs1005464 (*BMP2*)	Recessive (AA + AA vs. GG + GG+ AG +AG)	0.022*	4.06	1.22	13.47

Note: Poisson Regression was applied. The models were adjusted by age. PR means Prevalence Ratio.

* indicate statistical significance (p<0.05)

None of the studied SNPs were associated with the hypodivergent profile (p> 0.05).

## Discussion

Unraveling the genetic factors involved in facial development contributes to the improvement of malocclusion predictability, orthodontic treatment planning, and patient response management [10, 14]. In this study, we assess the potential impact of SNPs on vertical facial profile establishment. Seven SNPs in well-known genes involved in craniofacial development [8, 10, 14] and associated with skeletal bone physiology [17, 18, 22] were chosen. We reject the null hypothesis; some SNPs in candidate genes may increase the risk of hyperdivergent facial growth. This finding has significant clinical implications for the fields of facial morphology and orthodontics. By incorporating genetic testing for these SNPs into orthodontic diagnostics, we may improve the accuracy of treatment planning and provide customized interventions based on the patient’s genetic predispositions. Our study provides promising prospects for personalized orthodontic treatment planning, which can lead to better patient outcomes.

There are several parameters for assessing vertical growth patterns in cephalometric analysis. Over the years, diagnostic accuracy studies were performed; however, no consensus on which parameter is the most reliable was reached [23]. For this study, we adopted the measurement (SN-MP) recommended by the American Board of Orthodontics to diagnose patients as normodivergent, hyperdivergent, or hypodivergent. Interestingly, SN-MP is recognized as a more accurate indicator for determining the facial vertical growth pattern in the majority of populations [23, 24]. However, high or low values of SN-MP can be influenced by various conditions, such as maxillary or mandibular dentoalveolar height variations, anterior cranium base inclination, and abnormal directional growth of the mandibular condyle or ramus. Another limitation of our study was the use of two dimensional (2D) images. Three dimensional (3D) tomographic images can provide more detailed information about the specific regions, which may be affected by SNPs variations in development of the vertical profile.

*RUNX2 i*s prominently expressed in intramembranous and endochondral growth regions. In the mandibular condyle, RUNX2 plays an important role during the phase of cartilage matrix substitution for trabecular bone. In intramembranous growth regions, such as the maxillary sutures and mandibular ramus, RUNX2 is indispensable for the differentiation of pluripotent mesenchymal cells into osteoblasts, while also having a crucial function in mature osteoblasts by sustaining the expression of genes responsible for bone matrix proteins [16]. Some SNPs in *RUNX2* were previously investigated [8, 22, 25] and associated with anteroposterior skeletal malocclusions [8, 22]. In this study, the minor allele (A) and recessive genotype (AA) of the rs1200425 were associated with a hyperdivergent profile. The rs1200425 is an intronic variant that may induce aberrant mRNA splicing, which is a critical step in the posttranscriptional regulation. The recessive genotype (AA) has already been associated with a decrease in RUNX2 expression in bone tissue [22]. Interestingly, studies indicate that RUNX2 regulates intramembranous and endochondral growth differently. Some working groups [26, 27] developed specific transgenic mice to evaluate the impact of *RUNX2* gene deletion only in chondrocytes (exclusive cells of endochondral growth) and not in osteoblasts, and compared the results between mice with *RUNX2* gene deletion in both cells and wild-type mice. They demonstrated that RUNX2 is not involved in cartilaginous deposition but only in the substitution of the cartilaginous matrix for trabecular bone, which is performed by osteoblasts after vascular invasion into the cartilage. In animals with Runx2 deficiency, Vascular endothelial factor (Vegf) expression, the main protein for vascular invasion, is nearly completely absent, and there is not trabecular bone substitution into the cartilaginous zone, which becomes larger compared to animals with normal levels of RUNX2. Thus, the cartilaginous zone continues to increase, independent of the substitution into bone by osteoblasts. In the intramembranous growth region, which is exclusively dependent on osteoblasts, the bone deposition is significantly affected during RUNX2 deficiency [16]. Therefore, we assumed that the recessive genotype (AA) of the rs1200425 in *RUNX2* does not affect the endochondral growth of the mandibular condyle and the synchondrosis of anterior cranium base, which are mainly responsible for the downward displacement of the anterior face. However, the SNP may affect intramembranous growth regions, such as the mandibular ramus, which is responsible for the forward growth of the mandible, which may explain the association of this SNP with the hyperdivergent profile. However, the SNP may affect intramembranous growth regions, and therefore remodeling processes of the mandible, which may explain the association of this SNP with the hyperdivergent profile.

BMPs are a family of molecules in key positions of pathways that regulate craniofacial growth. BMPs are multi-functional factors involved in development, proliferation, and differentiation of mature osteoprogenitor cells into osteoblasts. An animal study showed that mutations in Bmp2 resulted in severe craniofacial anomalies [17]. In this study, we investigated the association between vertical profiles and two SNPs in the *BMP2* gene. The genotype AA of the rs1005464 in the *BMP2* gene was associated with an increased risk of a hyperdivergent profile development. This SNP was previously associated with mandibular retrognathism [8] and mesiodistal tooth size [28]. Not very much is known about the rs1005464 in the scientific literature, that makes it difficult to understand its specific molecular effects. Future research could provide valuable insights into the potential role of this SNP in bone development.

The literature supports that BMP2 induces osteoblast differentiation through RUNX2 [29]. The SNP-SNP interaction analysis aims to explore how the interplay between two or more SNPs influences phenotype expression [8, 9, 20]. In our study, a noteworthy synergistic effect was observed between rs1200425 in *RUNX2* and rs1005464 in *BMP2* SNPs. Specifically, individuals carrying recessive genotypes for both SNPs exhibited a higher risk of developing hyperdivergent facial profiles as compared to those with only one recessive genotype. When the combined effect of two or more genotypes is greater than the sum of the individual effects we define this as synergism. Despite these findings, the precise molecular mechanism underlying SNP-SNP synergism remains a subject of ongoing investigation.

We also investigated SNPs in *BMP4* and *SMAD6* genes due to their previous association with skeletal malocclusions [8]. BMP4 is well-known for its involvement in cell differentiation, bone development and the studied SNP rs17563 is involved in craniofacial phenotypes [30]. BMP4 signaling is complex, with potential cross-talks, including SMAD signaling. SMADs are crucial proteins in signaling pathways that regulate the transcription of Transforming Growth Factor β (TGF-β) gene family members. SMAD6, in particular, inhibits BMP signaling in the nucleus by interacting with transcription repressors. SMAD6 plays an essential role in regulating BMPs during endochondral growth [18]. In our study, we did not find a statistical association between the studied SNPs in *BMP4* and *SMAD6* and vertical profiles. Future researches may provide further insights into whether these SNPs may be associated with vertical profiles.

In conclusion, our results support the link between genes and the development of vertical facial growth patterns. Our results suggest that SNPs in bone development related genes—*RUNX2* and *BMP2* are associated with hyperdivergent facial profiles.

## Supporting information

S1 Data (XLSX)

## References

[1] Cai J, Min Z, Deng Y, et al. Assessing the impact of occlusal plane rotation on facial aesthetics in orthodontic treatment: a machine learning approach. BMC Oral Health. 2024;24(30):10.1186/s12903-023-03817-y doi: 10.1186/s12903-023-03817-y 38184528 PMC10771708

[2] Martins MV, Santos PRD, Carneiro DPA, Meneghim MDC, Menezes CCD, Vedovello, SAS. Impact of facial profile on young adults’ oral health-related quality-of-life item levels: A hierarchical analysis. Dental Press Journal of Orthodontics. 2021;26:e2120147. 34932709 10.1590/2177-6709.26.6.e2120147.oar PMC8690352

[3] Kusnoto B, Schneider BJ. Control of the vertical dimension. In Seminars in Orthodontics. WB Saunders. 2000; 6(1): 33–42.

[4] Enoki C, Telles CDS, Matsumoto MAN. Dental-skeletal dimensions in growing individuals with variations in the lower facial height. Brazilian dental journal. 2004; 15: 68–74. doi: 10.1590/s0103-64402004000100013 15322649

[5] Wishney M, Darendeliler MA, Dalci O. Craniofacial growth studies in orthodontic research—lessons, considerations and controversies. Australasian Orthodontic Journal. 2018; 34(1): 61–69.

[6] Cameron N, Schell L. Human growth and development. Academic Press. 2021. Eds.

[7] Cunha A, Nelson-Filho P, Marañón-Vásquez GA, de Carvalho Ramos AG, Dantas B, Sebastiani AM, et al. Genetic variants in ACTN3 and MYO1H are associated with sagittal and vertical craniofacial skeletal patterns. Archives of Oral Biology. 2019; 97: 85–90. doi: 10.1016/j.archoralbio.2018.09.018 30366217

[8] Küchler EC, Reis CLB, Carelli J, Scariot R, Nelson‐Filho P, Coletta RD, et al. Potential interactions among single nucleotide polymorphisms in bone‐and cartilage‐related genes in skeletal malocclusions. Orthodontics & craniofacial research. 2021; 24(2): 277–287. doi: 10.1111/ocr.12433 33068497

[9] Küchler EC, Reis CLB, Marañón-Vásquez G, Nelson-Filho P, Matsumoto MAN, Stuani MBS, et al. Parathyroid hormone gene and genes involved in the maintenance of vitamin D levels association with mandibular retrognathism. Journal of Personalized Medicine. 2021; 11(5): 369. doi: 10.3390/jpm11050369 34063310 PMC8147469

[10] Reis CLB, Matsumoto MAN, Baratto-Filho F, Scariot R, Stuani MBS, Romano FL, et al. Impact of genetic variations in the WNT family members and RUNX2 on dental and skeletal maturation: a cross-sectional study. Head & Face Medicine. 2023; 19(1): 26. 37400934 10.1186/s13005-023-00372-3 PMC10316614

[11] Oelschlaeger P. Molecular Mechanisms and the Significance of Synonymous Mutations. Biomolecules 2024, 14, 132. doi: 10.3390/biom14010132 38275761 PMC10813300

[12] Hersberger-Zurfluh MA, Papageorgiou SN, Motro M, Kantarci A, Will LA, Eliades T. Vertical growth in mono-and dizygotic twins: A longitudinal cephalometric cohort study. Orthod Craniofac Res. 2020; 23(2): 192–201. doi: 10.1111/ocr.12358 31746097

[13] Kirschneck M, Zbidat N, Paddenberg E, Reis CLB, Madalena IR, de Menezes-Oliveira MAH, et al. Transforming Growth Factor Beta Receptor 2 (TGFBR2) Promoter Region Polymorphisms May Be Involved in Mandibular Retrognathism. BioMed research international. 2022; 1503052. doi: 10.1155/2022/1503052 35757474 PMC9217526

[14] Reis CLB, Marañón-Vásquez GA, Matsumoto MAN, Baratto-Filho F, Stuani MBS, Proff P, et al. Single nucleotide polymorphisms in odontogenesis-related genes associated with tooth-size discrepancy. Australasian Orthodontic Journal. 2023; 39(1): 86–95.

[15] Di Pietro L, Barba M, Palacios D, et al. Shaping modern human skull through epigenetic, transcriptional and post-transcriptional regulation of the RUNX2 master bone gene. Sci Rep. 2021; 11(1): 21316. Published 2021 Oct 29. doi: 10.1038/s41598-021-00511-3 34716352 PMC8556228

[16] Takarada T, Nakazato R, Tsuchikane A, Fujikawa K, Iezaki T, Yoneda Y, et al. Genetic analysis of Runx2 function during intramembranous ossification. Development. 2016; 143(2): 211–218. doi: 10.1242/dev.128793 26657773

[17] Chen Y, Wang Z, Chen Y, Zhang Y. Conditional deletion of Bmp2 in cranial neural crest cells recapitulates Pierre Robin sequence in mice. Cell Tissue Res. 2019; 376(2): 199–210. doi: 10.1007/s00441-018-2944-5 30413887 PMC6467800

[18] Paulsen M, Legewie S, Eils R, Karaulanov E, Niehrs C. Negative feedback in the bone morphogenetic protein 4 (BMP4) synexpression group governs its dynamic signaling range and canalizes development. Proceedings of the National Academy of Sciences. 2011; 108(25): 10202–10207. 10.1073/pnas.1100179108 PMC3121836 21633009

[19] Little J, Higgins JP, Ioannidis JP, et al. STrengthening the REporting of Genetic Association Studies (STREGA): an extension of the STROBE statement. PLoS Med. 2009; 6(2): e22. doi: 10.1371/journal.pmed.1000022 19192942 PMC2634792

[20] Küchler EC, Tannure PN, Falagan-Lotsch P, Lopes TS, Granjeiro JM, Amorim LMF. Buccal cells DNA extraction to obtain high quality human genomic DNA suitable for polymorphism genotyping by PCR-RFLP and Real-Time PCR. Journal of Applied Oral Science. 2012; 20: 467–471. doi: 10.1590/s1678-77572012000400013 23032210 PMC3881822

[21] Küchler EC, Reis CLB, Silva-Sousa AC, Marañón-Vásquez GA, Matsumoto MAN, Sebastiani A, et al. Exploring the association between genetic polymorphisms in genes involved in craniofacial development and isolated tooth agenesis. Frontiers in Physiology. 2021; 12: 723105. doi: 10.3389/fphys.2021.723105 34539446 PMC8440976

[22] Olsson B, da Silva MJ, Lago C, et al. Single Nucleotide Polymorphisms in Runt-related Transcription Factor 2 and Bone Morphogenetic Protein 2 Impact on Their Maxillary and Mandibular Gene Expression in Different Craniofacial Patterns—A Comparative Study. Ann Maxillofac Surg. 2021;11(2): 222–228. doi: 10.4103/ams.ams_40_21 35265489 PMC8848693

[23] Ahmed M, Shaikh A, Fida M. Diagnostic performance of various cephalometric parameters for the assessment of vertical growth pattern. Dental press journal of orthodontics. 2016; 21:41–49. doi: 10.1590/2177-6709.21.4.041-049.oar 27653263 PMC5029315

[24] Rizwan M, Mascarenhas R, Hussain A. Reliability of the existing vertical dysplasia indicators in assessing a definitive growth pattern. Rev Latinoam Ortod Odontopediatría. 2011; 1–7.

[25] Jazaldi F, Soegiharto BM, Hutabarat AD, Soedarsono N, Auerkari EI. Runx2 rs59983488 polymorphism in class II malocclusion in the Indonesian subpopulation. Majalah Kedokteran Gigi. 2021; 54(4): 216–220.

[26] Qin X, Jiang Q, Nagano K, Moriishi T, Miyazaki T, Komori H, et al. Runx2 is essential for the trans differentiation of chondrocytes into osteoblasts. PLoS Genet. 2021; 16(11): e1009169. 10.1371/journal.pgen.1009169 PMC7728394 33253203

[27] Rashid H, Chen H, Javed A. Runx2 is required for hypertrophic chondrocyte mediated degradation of cartilage matrix during endochondral ossification. Matrix Biology Plus. 2021; 12: 100088. doi: 10.1016/j.mbplus.2021.100088 34805821 PMC8586806

[28] Gerber JT, dos Santos KM, Brum BK, et al. Odontogenesis-related candidate genes involved in variations of permanent teeth size. Clin Oral Invest. 2021; 25, 4481–4494. doi: 10.1007/s00784-020-03760-0 33651240

[29] Jang WG, Kim EJ, Kim DK, Ryoo HM, Lee KB, Kim SH, et al. BMP2 protein regulates osteocalcin expression via Runx2-mediated Atf6 gene transcription. J Biol Chem. 2012; 6;287(2): 905–15. doi: 10.1074/jbc.M111.253187 22102412 PMC3256879

[30] Küchler EC, de Oliveira Stroparo JL, Bitencourt Reis CL, et al. Oral Cleft Related-Genes may be Involved in Root Curvature of Maxillary Lateral Incisors. The Cleft Palate Craniofacial Journal. 2024;61(2):177–183. doi: 10.1177/10556656221121062 35979589

