# Supplementary material for: Single Nucleotide Polymorphisms in RUNX2 and BMP2 contributes to different vertical facial profile
Source: PLoS One. 2024 May 21;19(5):e0303551. doi: 10.1371/journal.pone.0303551 (PMC11108145; doi:10.1371/journal.pone.0303551)
Supplement: Attachment — Submitted filename: Response to Reviewers.docx [file pone.0303551.s002.docx]

**Response to Review**

**Manuscript ID PONE-D-24-05576**

Dear Dr. Isola,

I hope this message finds you well. I am writing to provide our response to the reviews received for our manuscript titled "Single Nucleotide Polymorphisms in RUNX2 and BMP2 Contributes to different vertical facial Profiles" submitted to PLOS ONE. We appreciate the time and effort invested by you and the reviewers in evaluating our work.

We believe that this new version of the manuscript adequately addresses the reviewers' concerns and is now ready for publication in PLOS ONE.

In this response letter, we have addressed each of the reviewers' comments and suggestions in detail. We have revised the manuscript accordingly, aiming to enhance the clarity, rigor, and overall quality of our findings. We are providing a marked-up copy of the manuscript that highlights changes made to the original version (with track changes). Besides, we make an unmarked version of the revised manuscript without tracked changes.

Thank you once again for considering our work for publication in PLOS ONE,

Journal requirements:

1) Please ensure that your manuscript meets PLOS ONE's style requirements, including those for file naming. The PLOS ONE style templates can be found at

[https://journals.plos.org/plosone/s/file?id=wjVg/PLOSOne_formatting_sample_main_body.pdf](https://journals.plos.org/plosone/s/file?id=wjVg/PLOSOne_formatting_sample_main_body.pdf#search/plos/_blank) and

[https://journals.plos.org/plosone/s/file?id=ba62/PLOSOne_formatting_sample_title_authors_affiliations.pdf](https://journals.plos.org/plosone/s/file?id=ba62/PLOSOne_formatting_sample_title_authors_affiliations.pdf#search/plos/_blank)

*ANSWER: We have carefully reviewed our manuscript to ensure compliance with all of PLOS ONE's guidelines, including those related to file naming.Thank you for your consideration.*

2. Please provide additional details regarding participant consent. In the ethics statement in the Methods and online submission information, please ensure that you have specified what type you obtained (for instance, written or verbal, and if verbal, how it was documented and witnessed). If your study included minors, state whether you obtained consent from parents or guardians. If the need for consent was waived by the ethics committee, please include this information.

*ANSWER: We provide comprehensive information on participant consent, thank you.*

3. Thank you for stating the following financial disclosure:

“This study was financed in part by the Coordenação de Aperfeiçoamento de Pessoal de Nível Superior - Brasil (CAPES) - Finance Code 001 and Alexander-von-Humboldt-Foundation (Küchler/Kirschneck accepted in July 4th, 2019). The São Paulo Research Foundation (FAPESP) financed individual scholarship (CLBR, process 2021/02704-1).”

Please state what role the funders took in the study.  If the funders had no role, please state: "The funders had no role in study design, data collection and analysis, decision to publish, or preparation of the manuscript."

If this statement is not correct you must amend it as needed. Please include this amended Role of Funder statement in your cover letter; we will change the online submission form on your behalf.

*ANSWER: We rewrite the financial disclosure, thank you.*

3. In the online submission form you indicate that your data is not available for proprietary reasons and have provided a contact point for accessing this data. Please note that your current contact point is a co-author on this manuscript. According to our Data Policy, the contact point must not be an author on the manuscript and must be an institutional contact, ideally not an individual. Please revise your data statement to a non-author institutional point of contact, such as a data access or ethics committee, and send this to us via return email. Please also include contact information for the third party organization, and please include the full citation of where the data can be found.

*ANSWER: The data underlying the results presented in the study are available from Department of Pediatric Dentistry, School of Dentistry of Ribeirão Preto, University of São Paulo (contact via dciops@forp.usp.br)*

5. Your ethics statement should only appear in the Methods section of your manuscript. If your ethics statement is written in any section besides the Methods, please delete it from any other section.

*ANSWER: We delete the ethics statement of another section, thank you.*

6. Please include your tables as part of your main manuscript and remove the individual files. Please note that supplementary tables should remain as separate "supporting information" files.

*ANSWER: We corrected this, thank you.*

7. Please include captions for your Supporting Information files at the end of your manuscript, and update any in-text citations to match accordingly. Please see our Supporting Information guidelines for more information: [http://journals.plos.org/plosone/s/supporting-information](http://journals.plos.org/plosone/s/supporting-information#search/plos/_blank).

*ANSWER: Thank you!*

Reviewer #1

Thank you for submitting your article. This study investigated the association between different vertical facial profiles and SNPs in RUNX2, BMP2, BMP4, and SMAD6. It is a good topic and well written paper. I feel that is appropriate information to the Plos One and I have just a few considerations to do:

1- Abstract.
Page 2, line 36. You said “American Board of Orthodontics (angle formed....)”. You don’t need this information in abstract (angle formed by the intersection of anterior cranium base and mandibular plane lines), only in MM.

*ANSWER: We corrected this, thank you*

2- Introduction.
Page 3, there are typos like: line 59, “strongly associated”; line 63: “have harmonious”; line 66: “involves interactions”. Please review entire paper.

*ANSWER: Sorry for this. We review the entire paper. Thank you.*

3- Material and methods.
Page 5, line 120: “American Board of Orthodontics”. Here you cant put that information from abstract.

*ANSWER: We corrected this, thank you*

4- Line 122: You said “two experienced orthodontists”, How many years?

*ANSWER: We inserted this information, thank you.*

5- Discussion.
Page 8, line 198: the same typos, “development contributes”, please review entire paper.

*ANSWER: Sorry for this. We review the entire paper. Thank you.*

6 - Page 8, line 203. “Once these SNPs are associated.............to the establishment of new preventative therapies.” Is this information the clinical relevance of your paper? If yes, please make this clear.

*ANSWER: We rewrite the first paragraph of discussion to clarify this point, thank you*

7- References.
Please insert references from 2024.

*ANSWER: We inserted new references from 2024, thank you.*

Reviewer #2:

 The present study aimed to investigate the association between different vertical facial profiles and SNPs in RUNX2, BMP2, BMP4, and SMAD6.

1. Abstract: the aim should be clearer. Which SNPs?

*ANSWER: We believe that if we will add the SNPs studied the aim will be extensive. We clarify the aim of another way, thank you.*

2. In the introduction and discussion, I suggest that the authors evaluate the possibility of including more recent articles (2022 to 2024) so that the current state of the art on the subject is represented.

*ANSWER: We inserted new references from 2024, thank you.*

3. In general, English should be revised. Some words are spelled incorrectly, or together. For exemple: Gernerally.

*ANSWER: Sorry for this. We review the entire paper. Thank you.*

4. The order of the session of materials and methods should be reviewed. Study size need to be one of the first subtopics.

*ANSWER: We corrected this, thank you*

5. The tables need to be sequentially numbered based on their order of appearance in the text.

*ANSWER: We corrected this, thank you*

6. I request authors to revise the titles of all tables to ensure they are self-explanatory and easily understandable.

*ANSWER: We corrected this, thank you*

7. How were the 7 SNPs chosen? This should be discussed in the discussion section.

*ANSWER: We rewrite the first paragraph of discussion to clarify this point, thank you*

8. I suggest that the authors include a paragraph with the clinical relevance of the study in the Discussion Session.

*ANSWER: We rewrite the first paragraph of discussion to clarify this point, thank you*

9. I kindly request that the authors carefully review the standards and guidelines set forth by the journal for their articles.

*ANSWER: We corrected this, thank you*
